# What are the safety risks for patients undergoing treatment by multiple specialties: a retrospective patient record review study

**DOI:** 10.1186/1472-6963-13-497

**Published:** 2013-11-28

**Authors:** Rebecca J Baines, Martine C de Bruijne, Maaike Langelaan, Cordula Wagner

**Affiliations:** 1 Department of Public and Occupational Health & EMGO Institute for Health and Care Research, Vrije Universiteit Medical Center (VUmc), Amsterdam, The Netherlands; 2 NIVEL, Netherlands Institute for Health Services Research, Utrecht, The Netherlands

**Keywords:** Patient safety, Hospital medicine, Medical error, Measurement, Adverse events, Epidemiology and detection

## Abstract

**Background:**

If multiple medical specialties are involved in treatment there is a danger of increasing risks to patient safety. This is due to the need for greater co-ordination and communication with other specialties, less emergency cover for individual sub-specialties, and a drop in general care and the overview of care. This study aims to determine if the number of medical specialties treating a patient is associated with the risk of experiencing harm during hospital admission.

**Methods:**

We performed a retrospective patient record review study using a stratified sample of 20 hospitals in the Netherlands. In each hospital 200 patient admissions were included. We related the occurrence of preventable adverse events and non-preventable adverse events to the number of specialties treating a patient through a stepwise multilevel logistic regression analysis.

**Results:**

Compared to patients treated by only one specialty, patients treated by three or more specialties had an odds ratio of experiencing an adverse event of 3.01 (95% CI 2.09 to 4.34), and an odds ratio of experiencing a preventable adverse event of 2.78 (95% CI 1.77 to 4.37). After adding characteristics related to the patient and the type of health care, the odds ratio for non-preventable adverse events decreased to 1.46 (95% CI 0.95 to 2.26), and for preventable adverse events to 2.31 (95% CI 1.40 to 3.81). There were no large differences found between the groups relating to the causes of preventable adverse events. However, in patients treated by three or more specialties, the greater number of preventable adverse events was related to the diagnostic process.

**Conclusions:**

The more specialties treating a patient the greater the risk of an adverse event. This finding became more pronounced for preventable adverse events than for non-preventable adverse events after corrections for the characteristics of the patient and their health care. This study highlights the importance of taking the number of specialties treating a patient into account. More research is needed to gain insight into the underlying causes of inadequate care when multiple specialties are required to treat a patient. This could result in appropriate solutions resulting in improvements to care.

## Background

Hospitals have become increasingly complex organisations. Scientific and technological progress has been followed by specialisation and further sub-specialisation of the medical profession. Increasing specialisation can have positive as well as negative effects on the care of patients. The care given is more specialised so there is more specific knowledge on, and experience of, specific diseases and treatment options. Specialisation in this way is often seen as a way to improve patient outcomes, especially in surgery [1,2]. On the other hand, increased specialisation may lead to inadequate care and increased risks to patient safety. This is due to an increased need for co-ordination and communication with other specialties, the fragmentation of care, less emergency cover for individual sub-specialties and a drop in general care and the overview of care. An example of how specialisation can lead to greater risks for patients is that doctors within a specific specialty seem to be biased towards diagnosing patients within their own domain [3]. The more specialisation and more specialties treating a patient thus may lead to improved care, but also to extra safety risks for patients. We do not know yet if, indeed, an increased risk exists, and if it does, how large the risk is for patients treated by multiple specialties. Previous research on, for example, the high risk associated with hospital handovers does suggest that an increased risk for patients treated by multiple specialties could exist [4,5].

**Figure 1:**
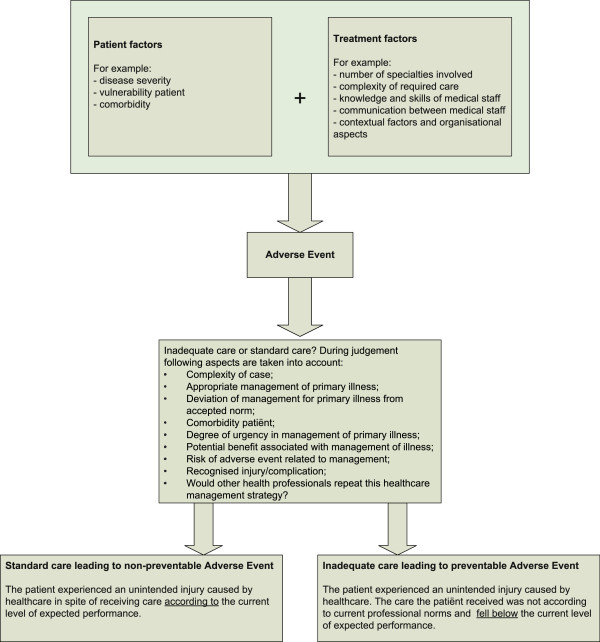
patient and treatment factors leading to preventable and non-preventable AEs.

In this article we explore if patients treated by more specialties are at a higher risk of experiencing harm during hospital admissions. A retrospective patient record review to assess adverse events (AEs) provides a unique opportunity to study the above mentioned questions. In this type of research entire patient admissions are reviewed to see if a patient experienced an AE. An AE is seen as an unintended injury that results in temporary or permanent disability, death or prolonged hospital stay, and is caused by healthcare management rather than by the patient’s underlying disease process [6,7]. The degree to which an AE is preventable is defined by judging if inadequate care had caused the adverse event. In other words if the care given fell below the current level of performance expected of practitioners or systems. The number of specialties treating a patient and the possible communication and co-ordination problems associated with this could have a role in inadequate care. AEs which could not be prevented are unintended injuries caused by health care in spite of receiving care according to the current level of expected performance (Figure 1). This study provides a first indication of whether the number of specialties treating a patient is associated with patients’ risk of experiencing harm during hospital admissions. If an association exists then we will explore the contribution made by inadequate care by comparing the preventable and non-preventable AEs.

## Methods

### Design and setting

We performed a retrospective patient record review study from 2009 to 2010 in 20 of the 93 Dutch hospitals [8]. These comprised, four university hospitals, six tertiary teaching hospitals and ten general hospitals. The sample was stratified for hospital type (university, tertiary teaching and general hospitals), representation of urban and rural settings in the samples were verified. Hospitals were eligible if they had at least 200 beds, an emergency room and an intensive care unit.

In each hospital, a stratified sample of 200 admissions from 2008 was selected. Fifty per cent of the records were from patients discharged from the hospital after more than 24 hours. The other fifty per cent was of patients who deceased in hospital. The admissions were selected randomly within these two strata. In our national studies, we chose to oversample hospital deaths in order to be able to assess the rate of preventable deaths [6]. Admissions to the psychiatric department, obstetrics department and admission of children aged less than one year, were excluded.

### Record review

The nursing, medical and, if available, outpatient record of the index admissions were reviewed by 39 nurses and 19 medical consultants drawn from the specialties surgery, internal medicine and neurology. Consultation with specialties, other than their own, was available, if needed, for the review of a patient record. The method of determining AEs was comparable to those of other international studies [7,9]. Firstly, a nurse screened the records by using 16 triggers indicating potential AEs, for example, re-admission or hospital acquired infection. During the review process additional data was gathered on the number of specialties involved in the treatment of the patient during the index-admission. The nurses were asked to note down which specialties were involved in the treatment of the patient. The nurses were able to choose from an extensive, though not exhaustive, list of 21 different specialties. These were: cardiology, surgery, dermatology, geriatrics, gynaecology, intensive care, internal medicine, paediatrics, ear, nose and throat, pulmonology, oncology, nephrology, neurosurgery, neurology, ophthalmology, orthopaedics, psychiatry, radiology, rehabilitation medicine, rheumatology and urology. If a specialty involved in treatment was not on this list, the nurses could also choose for the option “other” and write down the specialty. The specialties written down were: gastroenterology, endocrinology, vascular surgery, thoracic surgery, plastic surgery, anaesthesia for pain treatment, infectious diseases, haematology, dental medicine and cardiac surgery. The nurses were instructed to record a specialty as being involved in the treatment if they could find any evidence in the patient record of a consultation or an involvement in the treatment.

Admissions marked positive for at least one of the 16 triggers were reviewed further by a doctor. The presence and preventability of an AE was determined based on a standardised procedure. An AE was defined by three criteria:

1. an unintended injury.

2. resulting in a longer stay in hospital, a temporary or permanent disability, or death.

3. caused by healthcare management rather than the patient’s disease.

The medical reviewers took a decision on how far the AE could have been prevented based on a thorough analysis of the patient record by assessing, systematically, whether the care given fell below the current level of performance expected from practitioners or health systems. The cause of an AE, as well as the degree to which it could be prevented, were scored on a six-point likert scale and only counted as caused by health care or preventable if the score was four to six as is common to other studies of AEs [7,10].

AEs that occurred during the index hospital admission and were detected, either during the index admission, or subsequent admissions over the following 12-month period, were counted. In contrast to previous articles from our research group, AEs related to admissions within the 12 months preceding the index admission and detected during the index admission, were not counted for this article. This was because we had no information on that specific admission and the number of specialties involved in the treatment of the patient.

We linked the admissions included in the sample to the hospital administration database (Dutch Hospital Data) to obtain ICD9 diagnostic information.

### Exploring underlying mechanisms

We divided AEs into those which were non-preventable, having a preventability score of one to three, and those which were preventable, having a preventability score of four to six. To add structure to the implicit review process the reviewer’s judgement of inadequate care, and thus the presence of a preventable AE, was preceded by thirteen questions. These questions help the reviewers with their assessment of inadequate care, take into account the patient’s comorbidity and the complexity of the one or more diseases suffered. The care received by the patient is taken into consideration. We asked: Is the given care appropriate, deviant from the accepted norm, urgent, what is its potential advantage, what is the risk of an AE, is the injury a recognised complication and would other health care professionals repeat the care given? (Figure 1).

In this article we analyse preventable and non-preventable AEs to assess if inadequate care plays a role in AEs with patients treated by multiple specialties. Of course the difference between the two is often not black and white. However, it is important to seek out if in patients treated by multiple specialties the pathway leading to a preventable AE, or part of it, is different from the one leading to a non-preventable AE. We believe that the vulnerability of a patient, the complexity of a patient’s illness or illnesses, and the complexity of the treatment at hand, are all factors, amongst others, that may contribute to either preventable as well as non-preventable AEs (Figure 1). The main difference between both types of AEs however is that, in non-preventable AEs, the patient has received standard care and in spite of this suffered harm. In the origin of a preventable AE, however, inadequate care plays an important role, and at least in part, results in the patient suffering harm (Figure 1). For example, if a patient has had a previously unknown allergic reaction to a medication then this patient has received optimal care and in spite of this experienced a non-preventable AE. However, if this patient had previously had an allergic reaction to this medication and this was also written down in the patient’s record, then the AE is preventable. This patient has received inadequate care as, despite the allergy being written down in the patient’s record, the medication was given to the patient in question. When more caregivers are involved in the treatment during an admission, there may be a greater chance that information is missed.

When there are an increasing number of specialties involved in the treatment of a patient then we assess if the pathway of preventable AEs is different from non-preventable AEs. We achieve this in our analysis by correcting for factors representing the complexity of the patient and the complexity of the treatment. If the risk of preventable AEs and non-preventable AEs for different numbers of specialties treating a patient do not stay the same, this may indicate a difference in the risk of experiencing inadequate care.

If an AE was identified then a variety of questions were asked about it such as what are the clinical processes related to the AE. The medical reviewer could choose between the following processes: diagnostic, surgical, drug/fluid, medical procedure, other clinical management, discharge, and other. The doctors were also asked to select all the causes that contributed to the occurrence of the AE using the taxonomy of the Eindhoven Classification Model [11]. The model is based on the system approach to accident causation from Reason and the skill-rules-knowledge based framework of Rasmussen [12,13]. The main categories of the model are: human (related to deficits in knowledge, skills or rules of conduct); organisational (related to procedures, information transfer, culture and organisational decisions and priorities); technical (related to design, construction, software or material); patient-related (patient characteristics outside the control of health care professionals, for example comorbidity, communicative skills or ethnicity); and other causes.

### Statistical analysis

The number of specialties involved in the treatment of a patient was grouped into '1 specialty’, '2 specialties’ or '3 or more specialties’ for each patient in order to assess if patients with an increasing number of specialties also have an increasing risk of experiencing AEs.

Descriptive statistics on patient and admission characteristics by the number of specialties treating a patient were analysed using SPSS 20.0. After weighing for the sampling frame, the total study sample was representative of the total Dutch population of hospitalised patients. The sample weight was the inverse of the probability of being included in the sample owing to the sample design.

Furthermore, we assessed the association between the number of specialties involved in the treatment of a patient and the presence of preventable and non-preventable AEs through stepwise multilevel logistic regression analysis (MLwin 2.22). Multilevel analysis was used because the data had a hierarchical structure: patients (level 1) were clustered within hospital departments (level 2), and hospital departments were clustered within hospitals (level 3) [14]. The 2^nd^ order PQL estimation procedure was used. Variables were added to the model in order to correct for any possible patient and treatment factors influencing the association between the number of specialties involved in the treatment of a patient and the presence of preventable and non-preventable AEs. Model 1 was a naïve analysis with the number of specialties treating a patient as variable. Corrections were made for the stratified sampling method, with regard to deceased patients and the type of hospital. In model 2 corrections were made for the patient characteristics gender, age and the ICD9 main diagnostic group. The categories that were less common, that is less than three per cent, in our sample were pooled in other group. This left us with nine major categories and one other group. In model 3 we added the health care related characteristics that influence the type of care received in the hospital, such as the transfer to an intensive care unit during admission, surgery during admission, the length of admission and the urgency of admission. All the models were analysed for the outcome preventable AE as well as non-preventable AE, in order to assess the differences in the contribution of inadequate care between the numbers of specialties involved. After corrections we calculated intraclass correlation coefficients (ICCs), the ratio of the between group variance and the total variance. A higher ICC at the department level for instance means a smaller variance for preventable AE rates within the departments and a larger variance between departments.

We analysed the proportions of causes and clinical processes related to preventable AEs in order to assess further the underlying mechanisms.

We performed two sensitivity analyses. For the first sensitivity analysis, we added the Charlson comorbidity index (CI) to the model in model 2 to correct further for patient complexity. The CI is a comorbidity scoring system that includes weighting factors on the basis of the severity of a disease [15]. The CI could only be calculated for 18 out of the 20 hospitals.

The second sensitivity analysis was performed because intensive care was not always counted as a separate specialty, so that not all patients admitted to an intensive care unit had intensive care named as a separate specialty in their treatment. To account for the possibility that intensive care was not a new specialty for patients after they were transferred, we performed a second sensitivity analysis without counting intensive care as a separate specialty.

Interrater specific agreement statistics were calculated, that is the positive and negative agreement [16]. For the assessment of AEs the positive agreement was 63%, the negative agreement 87%. For assessment of the preventability of AEs, the positive agreement was 71% and negative agreement 76%.

## Results

In total 4,023 patient records were reviewed. Of these 27 patient records were excluded during analysis because they could not be linked to the hospital administration database and consequently had missing ICD9 main diagnostic group information. During the index admission 269 patients experienced a non-preventable AE, 191 a preventable AE. In total 2,117 patients were treated in only one specialty, 924 by two and 955 by three or more. After weighing for the stratified sample this amounts to 69% patients treated in one specialty, 19% in two and 12% in three or more. In this last group the maximum number of specialties treating a patient was 12 (one patient), but most patients in this group had three (487 patients), four (247 patients) or five (107 patients) specialties.

Patients treated by more than one specialty were older, were admitted for longer, were admitted more often urgently, underwent a surgical procedure less often, were admitted more often to an intensive care unit and were admitted more often to the department’s internal medicine and neurology (Table 1).

**Table 1:** Hospital and patient characteristics of the study samples for 1, 2 or 3 or more specialties

**Hospital and patient characteristics †**	**One specialty**	**Two specialties**	**Three or more specialties**	**Total**
Male sex %, n = 2051	49.3	50.5	52.5	49.9
Age in years, mean (SD)	57.7 (21.0)	64.3 (19.7)	66.6 (17.5)	60.0 (20.7)
Length of hospital stay in days, mean (SD/median)	4.6 (5.3/3.0)	8.3 (8.0/6.0)	16.2 (16.6/12.0)	6.7 (8.9/4.0)
Urgent admissions %, n = 2727	46.3	69.2	76.5	54.1
Surgery during admission %, n = 1336	48.5	36.6	30.0	44.1
Admission to an intensive care unit %, n = 640	3.8	14.4	23.4	8.0
Hospital departments, column %				
- Surgery, n = 703	21.6	25.1	18.4	21.9
- Cardiology, n = 421	12.4	11.9	6.5	11.6
- Internal medicine, n = 863	13.7	20.2	24.5	16.2
- Orthopaedics, n = 255	13.2	5.3	7.3	11.0
- Neurology, n = 366	5.1	10.9	18.0	7.7
- Lung diseases, n = 374	5.9	7.3	6.1	6.2
- Ear, nose and throat, n = 91	5.1	0.8	0.4	3.7
- Urology, n = 127	6.1	3.4	2.3	5.2
- Other, n = 796	10.3	2.6	2.5	13.2
ICD-9 diagnostic groups, column %*	12.6	10.9	11.8	
- Neoplasm’s, n = 649	12.6	10.9	11.8	12.2
- Nervous system and sensory organs, n = 105	3.7	1.9	2.5	3.3
- Circulatory system, n = 1051	17.9	27.6	27.7	20.8
- Respiratory system, n = 453	8.7	9.1	8.0	8.7
- Digestive system, n = 355	10.8	11.1	9.7	10.8
- Genitourinary system, n = 187	6.9	5.2	3.8	6.3
- Musculoskeletal system and connective tissue, n = 261	14.1	6.5	6.3	11.9
- Ill-defined conditions, n = 242	6.2	5.9	7.6	6.3
- Injury and poisoning, n = 320	8.7	11.1	12.5	9.5
- Other ‡, n = 374	10.2	10.5	9.9	9.3

†Hospital and patient characteristics are weighted for overrepresentation of deceased patients and university hospitals. N: actual numbers of cases, not weighted.

‡Other: includes smallest groups (3% and under): infectious and parasitic diseases; endocrine, nutritional, metabolic and immunity; blood and blood forming; mental; complications birth; skin and subcutaneous disease; congenital abnormalities; V-codes. n = actual number, not weighted.

Stepwise multilevel logistic regression analyses showed that the number of specialties treating a patient is associated with the risk of experiencing either a non-preventable AE or preventable AE as is shown in Table 2.

**Table 2:** Multilevel regression analysis for the association between patients with at least one non-preventable AE or preventable AE during an admission and the number specialties

**No. of specialties**	**No. of non-preventable**	Model 1* OR	Model 2* OR	Model 3* OR
	**AE§**	**(95% CI)**	**(95% CI)**	**(95% CI)**
One specialty (reference group) (n = 2117)	77			
Two specialties (n = 924)	52	1.54 (1.03 to 2.30)	1.51 (1.01 to 2.25)	1.19 (0.78 to 1.83)
Three or more specialties (n = 955)	106	3.01 (2.09 to 4.34)	2.90 (2.01 to 4.18)	1.46 (0.95 to 2.26)

	**No. of preventable**	Model 1* OR	Model 2* OR	Model 3* OR
	**AE§**	**(95% CI)**	**(95% CI)**	**(95% CI)**
One specialty (reference group) (n = 2117)	51			
Two specialties (n = 924)	55	2.15 (1.36 to 3.39)	2.21 (1.40 to 3.48)	2.15 (1.35 to 3.43)
Three or more specialties (n = 955)	77	2.78 (1.77 to 4.37)	2.88 (1.83 to 4.54)	2.31 (1.40 to 3.81)

*Model 1: naïve model, corrections for sampling strategy (overrepresentation deceased patients and university hospitals); model 2: model 1 + corrections for age, sex and ICD9 main diagnostic groups; model 3: model 2 + corrections for admission characteristics: admission to intensive care, length of stay, urgency of admission and surgery during admission. § = actual number, not weighted.

Patients treated by three or more specialties during admission had an odds ratio (OR) of 3.01 (95% CI 2.09 to 4.34) for experiencing a non preventable AE and an OR of 2.78 (95% CI 1.77 to 4.37) for a preventable AE. After corrections for patient characteristics (model 2) ORs for non-preventable AEs, as well as preventable AEs, barely changed. Finally, after adding corrections for the characteristics of the treatment (model 3) the association between treatment by three or more specialties and non-preventable AEs decreased to an OR of 1.46 (95% CI 0.95 to 2.26) and for preventable AEs the OR decreased to 2.31 (95% CI 1.40 to 3.81). The same pattern, though less pronounced, was found for patients treated by two specialties during their hospital admission.

After corrections the ICC estimates for preventable AEs at the hospital level were 1.97 and at the department level 11.2, indicating more variation at the department level than at hospital level.

Examples are given in Table 3 to illustrate in what way treatment by multiple specialties could have an influence upon the origin of preventable AEs.

**Table 3:** case descriptions of AEs

	
	**Case description**
1	Admission recent myocardial infarction, heart failure, fever and suspected pneumonia in delirium patient with chronic renal insufficiency. Four specialties were involved during this admission (cardiology, geriatrics, internal medicine (main specialty), neurology). Confusion in nursing staff because different specialties (geriatrics and general internal medicine) did not discuss treatment policies and both communicated conflicting medications to the nursing staff, resulting in extra intervention and treatment for the patient. The reviewer scored the preventability as more than likely.
2	Admission with stomach ache and constipation. Four specialties were involved during this admission (general surgery (main specialty), gynaecology, intensivist, gastroenterology). CTscan showed ileus of the small intestine. Operation however followed ten days later resulting in unfavourable postoperative course. Communication with radiologist in an earlier stage could have led to a faster diagnosis. The reviewer scored the preventability as more than likely.
3	Admission for epileptic incident. Only one specialty was involved during this admission (neurology). Status epilepticus a month earlier, however after this previous admission no maintenance medication was given due to insufficient consultation internal medicine by neurologist. Adverse event was noticed during following admission. Reviewer scored the preventability as strong evidence of preventability.

We analysed clinical processes and causes related to preventable AEs in order to assess further the underlying mechanisms of preventable AEs (Tables 4 and 5). In total in 154 preventable AEs human causes were found, in 51 organisational causes, in 59 patient-related causes and in 14 technical causes. After weighting the results for the stratified sample, the causes amounted, correspondingly, to 75.0% human, 21.4% organisational, 20.4% patient-related and 5.8% technical (Table 4). No large differences were found in the main causes for the number of specialties treating patients.

**Table 4:** Distribution of causes related to preventable AEs by the number of specialties

**Causes related to preventable AEs**	**Human†**	**Organisation†**	**Patient†**	**Technical†**
Number of specialties (row %)				
- One specialty, n = 54	75.0	20.0	14.5	7.1
- Two specialties, n = 57	78.3	25.0	39.1	8.7
- Three or more specialties, n = 80	72.0	24.0	20.0	0
Total, n = 191	75.0	21.4	20.4	5.8

†Percentages causes weighted for overrepresentation of deceased patients and university hospitals. n: actual numbers of causes, not weighted.

Reviewers could select more than one causal factor per AE.

Row percentages are given. For example the first row describes the distribution of causes related to all preventable AEs with one specialty treating a patient. Total percentages can add up to more than 100%, as reviewers could give more than one cause per AE.

**Table 5:** Distribution of clinical processes related to preventable AEs by the number of specialties

**Clinical process related to preventable adverse event n = 206**	**Diagnostic†**	**Surgical†**	**Medication†**	**Other clinical management†**	Other†*
Number of specialties (row %)					
- One specialty, n = 54	14.3	73.2	0.0	7.1	5.4
- Two specialties, n = 57	13.0	43.5	13.0	21.7	8.7
- Three or more specialties, n = 80	32.0	44.0	12.0	4.0	8.0
Total, n = 191	18.1	60.0	6.7	8.6	6.7

†Clinical process is weighted for overrepresentation of deceased patients and university hospitals.

*Including non-surgical interventions.

N = actual number, not weighted.

Row percentages are given. For example the first row describes the distribution of clinical processes related to all preventable AEs with one specialty treating a patient.

With regard to the clinical processes, more preventable AEs were related to diagnostic processes, in patients treated in multiple specialties - especially those treated in three or more - in comparison to those treated in just one. These figures were 32.0% as opposed to 14.3% (Table 5). 73.2% of the preventable AEs in patients treated in just one specialty were related to the surgical process that is the AEs occurred during surgery or within the postoperative period of 30 days. By contrast in patients with three or more specialties the figure was 44.0% (Table 5).

A sensitivity analysis adding the Charlson index to correct further for patient complexity in 18 hospitals had no effect and showed practically the same ORs, at that level, for no-preventable AEs as well as preventable AEs.

Sensitivity analyses excluding intensive care as a separate specialty showed the same pattern in association between adverse events and treatment in multiple specialties, only with lower ORs. Patients treated by three or more specialties had an OR of 1.95 (95% CI 2.33 to 5.19) for preventable AEs, after corrections for patient and health care related characteristics (model 3).

## Discussion

### General findings

The number of specialties treating a patient is associated with a patient’s risk of experiencing harm during a hospital admission. We found this variation for both preventable and non-preventable AEs. It was most pronounced in patients treated by three or more specialties. These variations were hardly explained by patient characteristics (age, sex, ICD9 major diagnostic group, Charlson index). They were explained in part by health care related characteristics (admission to intensive care, length of stay, urgency of admission and surgery during admission). This was more so for the non-preventable AEs than for the preventable ones. Thus after corrections for patient and health care characteristics the increased risk of harm for patients treated in multiple specialties stayed most visible in preventable AEs. This indicates a difference in that part of the pathway leading to preventable and non-preventable AEs since preventable AEs are related to inadequate care, whereas non-preventable AEs are related to limitations in today’s health care.

Our results indicate that inadequate care increases with the number of specialties involved in treatment independent of the complexity of the patient and treatment. However our data cannot confirm this is a causal relationship. After examining the causes of preventable AEs further we could not find evidence of, for example, more organisational or human-related causes in the different groups of numbers of specialties treating a patient. We did find more preventable AEs related to diagnostics in patients with three or more specialties, and more surgical AEs in patients with only one specialty.

### Other research

We did not find any previous studies combining the risk of experiencing AEs and preventable AEs and the number of specialties treating a patient. Links can be made to other areas of research looking at either increasing complexity in hospitals, or focussing on communication errors.

Higashi and colleagues found that, contrary to their expectations, the quality of care increased when the number of chronic conditions suffered by a patient increased [17]. They measured the quality of care by determining the deliverance of the percentage of care included in the indicators of quality care. This contrasts with our results, although these indicators of quality care did not concern outcome measures. Furthermore Nardi and colleagues discuss that complexity is far more than the co-existence of more than one medically diagnosed diseases [18]. Patient complexity involves the intricate interrelation between two or more systems, as for example illness, therapy, cognition, family, and behaviour [18]. From this perspective, patients treated by multiple specialties defines a part of patient and care complexity, but of course not all.

Delivering professional care to a complex patient who needs multiple specialties simultaneously or in a sequence is associated with an increased risk of preventable AEs. Communication failures are often mentioned as a common, if not the leading, cause of patient harm [19,20]. Problems in care due to poor communication between doctors seems likely to contribute often to preventable AEs [21,22]. Research on this topic focuses primarily on the communication related to teamwork in one group or setting [23]. Improving interpersonal communication during consultation, multidisciplinary handovers or multidisciplinary meetings may help. Applying Crew Resource Management (CRM) principles in order to develop a greater situational awareness and recognition of human factors could be of use in these situations. Introducing checklists or briefings may support the use of CRM principles to improve multidisciplinary teamwork. Communication is often just through writing when more specialties are involved, as for instance when a specialty is asked for a consultation. Often questions remain that cannot be pursued easily due to insufficient structure and information [24]. Verbal reports, in combination with written reports, have been shown to be the most effective [5]. Hierarchical differences, conflicting roles and conflicts also need to be taken into account when looking at communication failures [22].

Not only the communication between professionals, but also the communication between the patient and the different professionals may be associated with preventable AEs. Bartlett et al. found in 2008 that patients experiencing preventable AEs were three times more likely to have communication problems themselves [25]. Unfortunately, we did not collect data on all the patients’ communication capabilities, but this factor could also have influenced our results.

Patient risks due to increased specialisation could be reduced by improving communication with both professionals and the patient and by improving handovers. On the other hand, the call for more generalists such as hospitalists or geriatricians may also be seen as a way to decrease the risks. Although we did not find a direct link to patient safety, the introduction of hospitalists have demonstrated improved clinical efficiency and even a reduction of re-admissions [26], shorter hospital stays and a lower risk of mortality [27].

### Strengths and limitations

The strengths of this study are that complete patient admissions have been reviewed by independent qualified nurses and doctors in a structured and detailed manner to ascertain AEs, preventable AEs and underlying processes. In this manner, consecutive events can be studied, taking all available information into account. To our knowledge this is the first study linking AEs and the number of specialties treating a patient. This method cannot find out all the causes of AEs, as reviewers are dependent on information written down in the patient records [28]. This method also has some limitations concerning the number of specialties treating a patient. We could not account for the total number of individual specialists a patient came into contact with due to changing shifts but only the number of different specialties. Secondly, only different medical specialties involving doctors are taken into account in this study, other care givers that come into contact with patients as for example nurses or physiotherapists are not taken into account. Also, even though we have information on the number of specialties treating a patient, we do not have information on the specific contribution they made to the treatment of the patient. It is thus unclear if the specialist was simply consulted by telephone by the senior specialist, or if this specialist had actually seen the patient for a consultation, or if this specialist also had fully participated in the treatment of the patient during the whole admission. Lastly, we do not have information on at what moment the specialties were involved during the admission. In theory, more specialties could become involved after an adverse event has happened. However, looking at the nature of the adverse events, we expect this is less likely than other reasons for involving further specialties as described in our model (Figure 1).

## Conclusions

In conclusion a greater number of specialties treating a patient is associated with an increased risk of experiencing preventable AEs, which are related to inadequate care. More research is needed to gain insight into the underlying causes and any suitable solutions. We hope this study increases awareness of the fact that patients treated by multiple specialties have a higher risk of experiencing preventable AEs. This awareness can encourage the use of prevention strategies to avoid extra harm to this patient group. These include changes to the organisational structure, improved communication methods and methods to heighten an awareness of the situation.

### Ethical approval

The study protocol was reviewed and approved by the scientific committee of the EMGO + Institute, and by the ethical review board of the VU University Medical Center.

## Abbreviations

AE: Adverse event; CRM: Crew Resource Management.

## Competing interests

The authors declare that they have no competing interests.

## Authors’ contributions

RB: analysis and interpretation of data, drafting of the article; MdB: design, interpretation of data, critical revision of its intellectual content; ML: design, interpretation of data, critical revision of its intellectual content; CW: design, critical revision of its intellectual content, guarantor. All authors approved the final version.

## Pre-publication history

The pre-publication history for this paper can be accessed here:

http://www.biomedcentral.com/1472-6963/13/497/prepub
